# Obstructive Sleep Disorders in Down Syndrome’s Children with and without Lower Airway Anomalies

**DOI:** 10.3390/children8080693

**Published:** 2021-08-12

**Authors:** Mariska De Lausnay, Stijn Verhulst, Kim Van Hoorenbeeck, An Boudewyns

**Affiliations:** 1 Department of Pediatrics, Antwerp University Hospital, Drie Eikenstraat 655, 2650 Edegem, Belgium; stijn.verhulst@uza.be (S.V.); kim.vanhoorenbeeck@uza.be (K.V.H.); 2 Laboratory of Experimental Medicine and Pediatrics (LEMP), Antwerp University, 2610 Antwerpen, Belgium; 3 Department of Otorhinolaryngology, Antwerp University Hospital, Drie Eikenstraat 655, 2650 Edegem, Belgium; an.boudewyns@uza.be

**Keywords:** Down syndrome, obstructive sleep apnea, airway malacia, endoscopy

## Abstract

(1) Background: Obstructive sleep apnea (OSA) and lower airway anomalies are both highly prevalent in children with Down syndrome (DS). However, little is known on the interaction between both. We aim to investigate the co-occurrence of OSA (defined as obstructive apnea/hypopnea index (oAHI) ≥ 2/h) and lower airway anomalies in children with DS and explore their impact on OSA severity and treatment outcome. (2) Methods: Retrospective analysis of data from airway endoscopy and polysomnography (PSG) in a cohort of children with DS. (3) Results: Data on both lower airway evaluation and PSG were available for 70 patients with DS. Our study population was relatively young (mean age 3.5 years), not obese and presented with severe OSA (mean oAHI 13.1/h). Airway anomalies were found in 49/70 children (70%), most frequently laryngomalacia, tracheomalacia or a combined airway malformation. In the remaining 21 cases (30%), endoscopy was normal. A comparison between both groups showed a similar distribution of gender, age and BMI z-scores. The prevalence of OSA was not significantly higher in DS patients with airway anomalies (89.6% vs 71.4%, *p* = 0.078). Additionally, OSA severity or treatment choice (conservative, upper airway surgery or CPAP) were not significantly different. Follow-up data (available for 49/70 patients) showed a significant improvement of OSA in both groups. There is a not significant tendency to more patients with persistent OSA among those with lower airway anomalies (34.3% vs 7.1%, *p* = 0.075). (4) Conclusions: We found no significant differences in OSA severity, treatment choice or outcome between children with DS with and without lower airway anomalies. Further studies should investigate the role of DISE-directed treatment and compare the outcome of different treatment modalities in larger patient groups.

## 1. Introduction

Sleep disorders are a prevalent health problem in children with Down syndrome [1,2]. Previous studies demonstrated that more than half of the children with DS have obstructive sleep apnea (OSA), documented by full night polysomnography (PSG) [1]. This high prevalence was found across all age groups (0–18 years) [1]. Since parent-reported OSA signs such as snoring do not correlate well with PSG findings [2], it is advised by the American Academy of Pediatrics that all children with DS are referred for PSG at the age of four years or earlier in case of suggestive symptoms.

Obstructive sleep apnea is characterized by recurrent episodes of complete or partial upper airway obstruction, disrupting normal ventilation and sleep continuity [3]. This usually results from a combination of upper airway narrowing (e.g., by adenotonsillar hypertrophy) and inadequate compensation for a decrease in upper airway neuromuscular tone [4]. Undiagnosed and/or untreated OSA may cause a wide range of comorbidities, such as behavioral and cognitive problems, failure to thrive and pulmonary hypertension [1,2]. The choice for a specific treatment regimen depends on many factors, such as the site of upper airway obstruction (often determined by a drug-induced sleep endoscopy), OSA severity, associated comorbidities and the patient and parental preferences. Treatment options for pediatric OSA are usually classified as either surgical (e.g., adenotonsillectomy, tongue base surgery, supraglottoplasty or maxillofacial surgery) or non-surgical (medical treatment, oral appliance therapy, myofunctional therapy, positional therapy and non-invasive ventilation such as continuous positive airway pressure (CPAP)) [4].

Factors contributing to OSA specifically in DS are craniofacial abnormalities (midfacial and mandibular hypoplasia, a narrow nasopharynx, relative macroglossia and adenotonsillar hypertrophy), hypotonia, and a tendency to obesity [1]. Airway anomalies such as laryngo- and tracheomalacia are also more common in children with DS compared to controls: Retrospective studies show the presence of one or multiple airway anomalies in over 70% of patients with DS who underwent endoscopic evaluation, which is significantly higher than in control populations [5,6,7]. Few data are available on the impact of these lower airway anomalies on OSA severity and treatment outcome in children with DS (where so many different factors contribute to the development of OSA); however, it seems plausible that these children present with more severe OSA, lower saturation, and/or more treatment failure [8].

The following three hypotheses were explored in this paper: (1) Is OSA more prevalent in DS children with airway anomalies compared to those without? (2) Is OSA severity different between DS children with/without lower airway anomalies? (3) Are there any differences in outcome of OSA treatment between DS children with/without lower airway anomalies?

## 2. Materials and Methods

This is a single center, retrospective study, where we collected data concerning lower airway endoscopy (dating from April 2011 to July 2020) and sleep studies from patient files. We confined inclusions to children and adolescents with DS aged 0–18 years.

We set up a database of patients with DS who underwent lower airway evaluation by a laryngo-and/or bronchoscopy and included the reason for endoscopic evaluation and the outcome. All direct laryngoscopies (flexible and rigid endoscopy of the upper and lower airway up to the level of the mainstem bronchi) and flexible bronchoscopies (including the tracheobronchial tree) were performed under general anesthesia with the child spontaneously breathing. Direct laryngoscopy was performed by pediatric ENT surgeons, flexible bronchoscopy was performed by pediatric pulmonologists.

For each child in this database, we searched if a PSG was performed. When multiple PSGs were conducted, we only used the first “baseline” PSG (before starting any treatment) and the first follow-up PSG (after initial treatment). All full overnight PSGs were performed at the Pediatric Sleep Disorders Center of the Antwerp University Hospital. The following variables were continuously measured and recorded by a computerized polysomnography (Brain RT, OSG, Rumst, Belgium): electroencephalography, electro-oculography, electromyography of anterior tibialis and chin muscles; and electrocardiography. Respiratory effort was measured by respiratory inductance plethysmography and oxygen saturation by a finger probe connected to a pulse oximeter. Airflow was measured by means of nasal pressure cannula and thermistor, and snoring was detected by means of a microphone at the suprasternal notch. Children were also monitored on audio/videotape using an infrared camera. Polysomnograms were manually scored by certified technicians according to international guidelines [9].

We collected data about PSG and sleep quality, such as total time in bed (TIB), total sleep time (TST), sleep latency (SL) and sleep efficiency (SE). We registered the number of both central and obstructive apneas and hypopneas and subsequently calculated the obstructive apnea-hypopnea index (oAHI). This is the number of obstructive apneas and hypopneas per hour of sleep (mixed apneas were very rare). A diagnosis of OSA in children is made by an oAHI ≥ 2/h (mild OSA: 2 ≤ oAHI < 5/h, moderate OSA: 5 ≤ oAHI < 10/h, severe OSA: oAHI ≥ 10/h) [10]. The effect of sleep disordered breathing on oxygen saturation was investigated by comparing the mean and minimal saturation during PSG, as well as the oxygen desaturation index (ODI). This is the average number of desaturation episodes (of ≥3%) per hour [9] and was only described in the more recent PSGs dating from 2012. For each patient, the choice of initial treatment was documented. In a large proportion of children diagnosed with OSA in this study population, a drug-induced sleep endoscopy (DISE) procedure was also performed to assess the pattern of upper airway obstruction. Treatment decisions were made based upon DISE findings (when available) and after multidisciplinary discussion of the findings as described earlier [1]. Treatment efficacy was evaluated by a repeat PSG. This second PSG was typically performed 3 months after a surgical intervention. Treatment outcomes were classified as follows: persistent OSA (defined as oAHI ≥ 5/h), successful treatment (oAHI 2.0–4.9/h), or cure (oAHI < 2/h).

The statistical analyses were conducted in SPSS (version 27). Comparing sexes and treatment regimen in both groups was done by Chi square test. Comparing continuous variables was done by independent samples t-test when there was a normal distribution or Mann-Whitney U test when non-parametric testing had to be used (for example, when comparing oAHI and ODI).

This study was approved by the Ethics committee of our institution (Antwerp University Hospital, Edegem, Belgium).

## 3. Results

### 3.1. All Patients with DS

During the study period, 76 pediatric patients from our DS cohort underwent a lower airway evaluation. In 70 of these children, data from one or more full night PSGs were available. These patients (with both lower airway evaluation and one or more PSGs) constitute our study population.

Sixty-four percent were boys. Mean age at time of lower airway evaluation was 3.2 years (SD 3.5 years). Sixty-two patients were younger than 6 years, six patients were between the age of 6 to 13 years and only two were 13 or older. As main indications for laryngo- and/or bronchoscopy, we note recurrent lower airway infections in 40%, stridor in 23% and chronic cough or noisy breathing in 21.4%. Other reasons were for example persistent infiltrates on lung imaging and respiratory failure.

**Figure 1:**
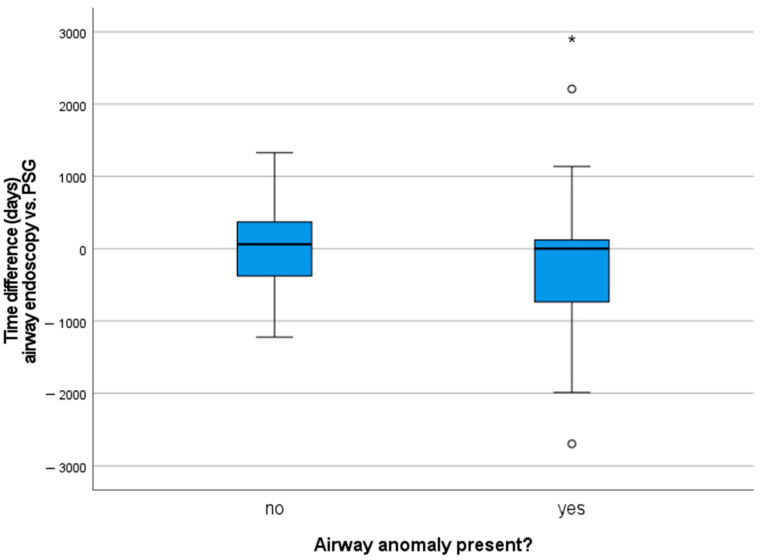
Comparison of the time difference between airway endoscopy and first PSG (in days). Outliers are displayed as ° or * for values deviating more than 1.5 or 3 interquartile range, respectively.

At the time of their first PSG, patients had a mean age of 3.5 years (age range one month to 14 years, SD 3.1 years) and were not obese (mean BMI z-score 0.33, SD 1.64). Half of the children underwent airway endoscopy prior to PSG, the other half after a sleep study was performed (see Figure 1, a positive value means that the children first had a PSG). This is a reflection of the clinical approach and retrospective analysis of the data: PSGs are performed around the age of four years or earlier based on clinical signs of OSA or airway problems.

Quality of PSG was considered good with a mean total sleep time (TST) of 530 min or 8.8 h and a sleep efficiency (SE) of 81%. Snoring was present in 44% of patients. A diagnosis of OSA was made in 84.1% (58/70) of patients, with severe OSA (defined as an oAHI ≥ 10/h) representing the largest part (48.6% of all patients). Mean oAHI was 13.1/h (range 0.1 to 70/h, SD 13.9/h). Mean saturation during the PSG was on average 95.1% for all investigated patients (range 89 to 98.9%, SD 2.4%) and minimum saturation 84.9% (SD 5.9%). For 63/70 patients, an oxygen desaturation index (ODI) was measured, with a mean of 5.6/h (range 0 to 24.1/h, SD 5.7/h).

Twenty patients (29%) received no or conservative therapy (i.e., pharmacological, position trainer) due to a normal PSG (*n* = 8) or mild to moderate OSA (*n* = 12). One child with moderate OSA (oAHI 9.2/h) was left untreated because of parental refusal to treatment. Thirty-four children (49.3%) were treated surgically: adenoidectomy (*n* = 6), tonsillectomy (*n* = 6), adenotonsillectomy (*n* = 18), supraglottoplasty (*n* = 3), maxillofacial surgery (*n* = 1). Nine patients already had previous upper airway surgery (e.g., repair of choanal atresia or tonsillectomy because of recurrent throat infections). Additionally, four children underwent surgery in the context of their airway anomaly: tracheoplasty (*n* = 1), cricotracheal resection (*n* = 1), aortopexy (*n* = 1), and repair of a vascular ring (*n* = 1). Fifteen patients (21.7%) were started on CPAP therapy. One patient was lost-to-follow-up after baseline PSG.

### 3.2. Comparison of DS Patients with and without Airway Anomalies

In our study population, airway anomalies were documented in 70% (49/70) of children: laryngomalacia (*n* = 12), tracheomalacia (*n* = 14), bronchomalacia (*n* = 4), subglottic stenosis (*n* = 2), tracheal bronchus (*n* = 1), and multiple anomalies (*n* = 16). All of the remaining 21 patients (30%) had a normal endoscopic evaluation of the lower airways.

**Figure 2:**
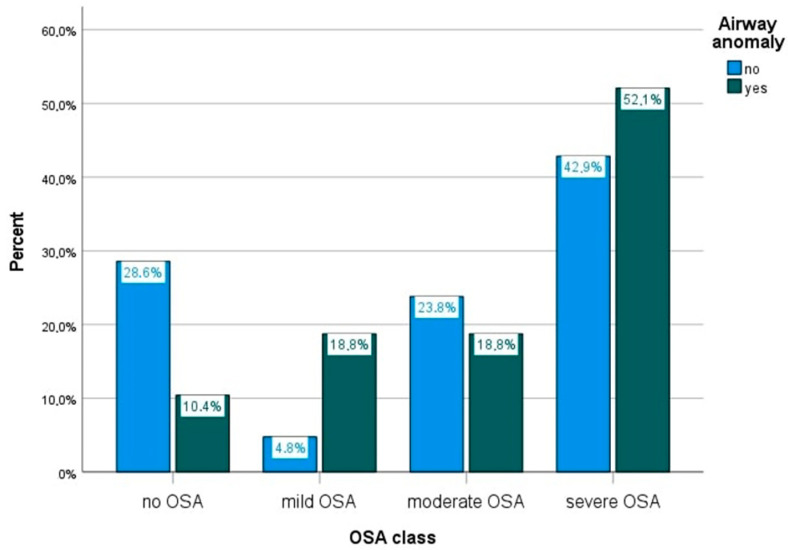
Comparison of OSA severity between the patients with and without airway anomalies.

In the groups with and without airway anomalies, there was a similar gender distribution with a male preponderance (*p* = 0.785). Children with a normal airway endoscopy had a better TST and SE. Previous upper airway surgery was performed in six patients with a lower airway anomaly (12.8%) and three patients without (15%; *p* = 0.806). PSG was requested at a later age in the subgroup with lower airway anomalies; however, not significantly (at 3.8 years compared to 2.7 years in the group with a normal airway, *p* = 0.739). There was no difference in presence of snoring during PSG (*p* = 0.659). A diagnosis of OSA was made more often in children with lower airway anomalies (in 89.6% compared to 71.4% in children without airway anomalies; *p* = 0.078) (see Figure 2). OSA severity (based on oAHI, OSA classification, mean saturation, minimum saturation, ODI) did not differ significantly between groups. The choice of treatment regimen was also similar. See Table 1 for a more detailed overview.

### 3.3. Comparison at Follow-Up PSG

For 49/70 patients, data of a follow-up PSG were available. The time interval between baseline and follow-up PSG was on average 10 months (range one to 30 months, SD 8 months), at a mean age of 4.4 years. After a surgical intervention, follow-up sleep study is usually performed after three to four months. Thirty-five children (71.4%) belong to the group with airway anomalies and fourteen (28.6%) to the group without. The mean age at time of follow-up PSG was not significantly different (4.8 years in children with lower airway anomalies compared to 3.4 years without; *p* = 0.569). TST and SE were no longer significantly different between the two groups. Despite a higher mean oAHI (7.7/h compared to 2.3/h, *p* = 0.499) and ODI (4.0/h compared to 2.5/h; *p* = 0.558) in the group with lower airway anomalies, none of the parameters concerning OSA severity differed significantly between the two groups. The outcome after initial OSA treatment (i.e., persistent OSA, successful treatment or cure) also showed no statistically significant differences (*p* = 0.139). Persistent OSA was observed in 34.3% (12/35) of children with DS and lower airway anomalies, compared to 7.1% (1/14) in the group without (*p* = 0.075). For more details, see Table 2.

When comparing the PSG parameters before and after treatment of the entire study population (*n* = 49), there is a significant improvement in mean oAHI, from 16.1/h to 6.1/h (oAHI difference −10/h; *p* < 0.001). This improvement was also significant in both subgroups separately. There was also a significant change in ODI (overall −2.3/h, *p* = 0.029), but for the subgroup with lower airway anomalies this was not significant (as determined by paired samples t-test). There were small but statistically significant improvements in mean saturation (overall +1%, *p* = 0.006) and minimum saturation (overall +3%, *p* = 0.001).

## 4. Discussion

In this study, we investigated the impact of lower airway anomalies on the presence, severity, and treatment outcome of OSA in children with DS. We expected that lower airway anomalies would adversely affect sleep disordered breathing, given their predisposition to airway collapse, chronic inflammation, and infection (sometimes needing positive expiratory pressure or CPAP therapy). However, the data from our study population could not support this hypothesis. A higher number of patients with airway anomalies was diagnosed with OSA (89.6% compared to 71.4%) but this difference was not statistically significant. In addition, OSA severity was not different between DS patients with or without airway anomalies. Finally, we observed a not statistically significant trend to more persistent OSA (defined as oAHI ≥ 5/h) at follow-up PSG in children with airway anomalies (in 34.3% compared to 7.1% in the group without *p* = 0.075). We suspect that these rather minor differences between the two groups can be explained by the multifactorial origin of OSA in DS, where the concurrence of risk factors is perhaps more important than one specific comorbidity. More specifically, we point out the combination of the craniofacial abnormalities, adenotonsillar hypertrophy and distinct hypotonia. At a later age, obesity becomes a considerable additional risk factor (unfortunately, the number of adolescent patients in our cohort was too small to investigate this factor).

Our findings may be influenced by the limited sample size. Baseline PSG data were available for 70 patients and a follow-up PSG only for 49/70 patients, making it a preliminary study. In addition, the number of patients in each treatment group (surgical, non-surgical, CPAP) was too small to perform statistical analysis with the aim to investigate the of a specific treatment modality.

An import sidenote is that four patients underwent surgery in the context of their airway anomalies (such as tracheoplasty), which may also have an effect on OSA. Another important consideration is the variable tolerance of CPAP therapy. For this study, we used data from the first follow-up sleep study, which in some cases was the titration night. As to be expected, these PSGs often show very good outcomes on the oAHI and saturation. About one-third of patients who were started on CPAP; however, did not tolerate CPAP for long-term usage and ceased therapy after a few months so that another PSG (with CPAP) was not available for comparison.

Despite these limitations, we conclude that (given the high prevalence of lower airway anomalies in this population [5,6,8]), the threshold for complete airway evaluation should be lower than in the general population (especially when other symptoms such as chronic cough, recurrent wheeze or stridor are present). A thorough pulmonary assessment of these children is still of utmost importance. Specifically for OSA, future studies should investigate the role of DISE-directed treatment and compare the outcome of different treatment modalities in larger patient groups and with more long-term follow-up data. Recent studies investigated the pattern of upper airway obstruction in children with DS and the outcomes of DISE-directed treatment. It would be of interest to investigate in a large group of patients, whether the outcome of DISE-directed treatment is different in children with DS and lower airway anomalies, which factors are associated with these outcomes and what the effect is at short and long-term. Hyzer et al. concluded that the pattern of upper airway collapse as identified through DISE, is different in surgically naïve children with obesity compared to those with DS. These authors recommend DISE in surgically naïve children with DS to guide treatment [11]. Akkina et al. also concluded that DISE-directed surgery may be beneficial for children with DS and OSA [12]. Raposo et al. concluded that children with DS have higher obstructive scores during DISE at the level of the tongue base and supraglottis and also an overall higher obstructive score [13].

Lastly, we noticed that snoring was only present in 42.9% of all patients, while there is a prevalence of OSA in 82.9% in this DS cohort. This confirms that the absence of typical symptoms is insufficient to rule out OSA in patients with DS. We; therefore, fully agree with the recommendation to screen all children with DS at a young age, irrespective of parent-mentioned obstructive breathing during sleep.

## Figures and Tables

**Table 1:** Patient characteristics, PSG markers, and choice of treatment in children with DS; compared between the group with and without lower airway anomalies.

	Airway Anomaly *n* = 49	Normal Airway *n* = 21	Comparison (*p*-Value)
Patient characteristics			
Sex (% boys)	32 (65.3%)	13 (61.9%)	0.785
BMI z-score	0.43	0.10	0.501
Mean age endoscopy (years)	3.3	2.9	0.158
Mean age PSG (years)	3.8	2.7	0.739
PSG markers			
TST (min)	507	584	**0.003**
SE (%)	78.3	87.0	**0.004**
Snoring (%) *	22 (45.8%)	8 (40%)	0.659
oAHI (/h) *	14.5	10.1	0.246
OSA present (%) - mild - moderate - severe	43 (89.6%)	15 (71.4%)	0.078
	9 (18.8%)	1 (4.8%)	0.157
	9 (18.8%)	5 (23.8%)	
	25 (52.1%)	9 (42.9%)	
Mean sat (%)	95.1	94.9	0.766
Min sat (%)	85.2	84.2	0.535
ODI (/h) *	6.2	4.2	0.579
Treatment:			0.544
- conservative - surgical - CPAP	12 (25%)	8 (38.1%)	0.270
	25 (52.1%)	9 (42.9%)	0.481
	11 (22.9%)	4 (19%)	0.720

Abbreviations: BMI = body mass index, PSG = polysomnogram, TST = total sleep time, SE = sleep efficiency, oAHI = obstructive apnea/hypopnea index, sat = saturation, ODI = oxygen desaturation index, CPAP = continuous positive airway pressure. * Missing values: for snoring 2/70; oAHI 1/70; for ODI 7/70; for treatment 1/70. Significant associations are shown in bold. Only valid percentages are shown.

**Table 2:** Comparison of the results from the first follow-up PSG after treatment between the group with and without lower airway anomalies.

	Airway Anomaly *n* = 35	Normal Airway *n* = 14	Comparison (*p*-Value)
Age 2nd PSG (years)	4.8	3.4	0.569
Time difference between PSGs (months)	10.1	10.3	0.513
TST (min)	508	580	0.064
SE (%)	80.2	85.6	0.215
oAHI (/h)	7.7	2.3	0.499
Mean sat (%)	96.3	95.7	0.250
Min sat (%)	87.9	87.6	0.811
ODI (/h) *	4.0	2.5	0.558
Result initial treatment:			0.139
- persistent OSA (oAHI ≥ 5/h) - success (oAHI 2–4.9/h) - cure (oAHI < 2/h)	12 (34.3%)	1 (7.1%)	0.075
	6 (17.1%)	4 (28.6%)	0.442
	17 (48.6%)	9 (64.3%)	0.360

Abbreviations: PSG = polysomnogram, TST = total sleep time, SE = sleep efficiency, oAHI = obstructive apnea/hypopnea index, sat = saturation, ODI = oxygen desaturation index. * Missing values for ODI: 3/49. Only valid percentages are shown.

## Data Availability

The datasets generated during and/or analyzed during the current study are available from the corresponding author on reasonable request.
